# A cross-sectional study on online food delivery applications (OFDAs) in the United Arab Emirates: use and perceptions of healthy food availability among university students

**DOI:** 10.1017/jns.2024.21

**Published:** 2024-10-10

**Authors:** Leila Cheikh Ismail, Tareq M. Osaili, Bayan Shanan, Dana Rashwan, Hulya Merie, Leen Rishan, Salam Al Shamma, Zaina AlRamahi, Sheima T. Saleh, Maysm N. Mohamad, Asma’ O. Taybeh, Rameez Al Daour, Sadi Taha, Lily Stojanovska, Ayoub Al-Jawaldeh, Ayesha S. Al Dhahei

**Affiliations:** 1 Department of Clinical Nutrition and Dietetics, College of Health Sciences, University of Sharjah, Sharjah, UAE; 2 Nuffield Department of Women’s & Reproductive Health, University of Oxford, Oxford, UK; 3 Research Institute for Medical and Health Sciences, University of Sharjah, Sharjah, UAE; 4 Department of Nutrition and Food Technology, Faculty of Agriculture, Jordan University of Science and Technology, Irbid, Jordan; 5 Department of Nutrition and Health, College of Medicine and Health Sciences, United Arab Emirates University, Al Ain, UAE; 6 Department of Nutrition and Food Processing, Faculty of Food Processing, Al-Huson University College, Al-Balqa Applied University, As-Salt, Jordan; 7 Institute for Health and Sport, Victoria University, Melbourne, Australia; 8 Regional Office for the Eastern Mediterranean (EMRO), World Health Organization (WHO), Cairo, Egypt

**Keywords:** Consumers’ perceptions, Food choices, Food delivery application, Healthy food availability, Online food environment

## Abstract

Online food delivery applications (OFDAs) have seen a surge in popularity during the COVID-19 Pandemic, particularly among young adults. This study aimed to assess the use of OFDAs and the perception of food healthiness and safety among university students in the United Arab Emirates (UAE). A web-based cross-sectional study was conducted among university students in the UAE via snowball sampling (n = 1096). Sociodemographic characteristics, OFDAs usage, and perceptions toward food healthiness and safety were investigated. Chi-square analysis was used to determine the association between categorical variables and healthy food choices. Mann–Whitney *U* and Kruskal–Wallis *H* tests were used to determine differences between different groups and perception statements. Over half (52%) of the students were frequent users of OFDAs with fast food being the most popular choice (88.4%). Older participants, those living in the university dorms, and those with higher allowances used the OFDAs more frequently (P < 0.05). Price (78.0%) and food appearance (65.7%) had the highest impact on food selection. Most subjects (69.3%) reported looking for healthy food but were worried about affordability (43.1%) and taste (27.1%). Most participants (57.8%) agreed that OFDAs increased their appetite and food intake. Participants believed that having a hygiene rating system in OFDAs would give them the chance to make informed decisions (82.3%). Unhealthy food options were popular among university students. The study highlights the need to provide more affordable and appealing healthy food options and suggests that improved nutrition information and hygiene standards could help to promote healthy food choices among university students.

## Introduction

Smart technology and mobile applications have become a crucial part of people’s everyday life. This led to the online system’s growth, impacting our conventional ways of dealing with daily tasks and activities and significantly impacting the retail industry.^(1)^ The concept of digital food environments refers to the online contexts in which food and nutrition information and services are provided and disseminated. These environments encompass a variety of digital mediums including, but not limited to, social media, digital health promotion interventions, digital food marketing, and online food retail platforms.^(2)^ Online food delivery applications (OFDAs) have become increasingly popular in recent years.^(3,4)^ According to global statistics, the number of OFDAs users increased by about 75% from 2019 to 2022, accounting for more than 1,850 million users globally compared to around 1,000 before the pandemic. This is expected to grow even further in the coming years.^(5)^ These digital platforms, allow users to browse menus, place orders, and track deliveries in real-time, all from their smartphones or other mobile devices.^(6)^

The increasing popularity of OFDAs is a reflection of the transformation in the digital food environment, which has led to the emergence of a range of digital platforms, including food delivery apps, online grocery stores, and meal kit delivery services.^(7)^ Both consumers and businesses have benefited greatly from these services. The convenience of ordering food from the comfort of one’s home or office has led to an increase in the usage of these apps.^(3)^ By accessing a larger audience online and enhancing their operational efficiency and delivery procedure, these apps have helped businesses like restaurants grow their customer base and revenue.^(8)^ From a public health perspective, while OFDAs have brought about many advantages for consumers, there are concerns about how the use of these apps can affect public health and eating patterns^(9)^ as well as the environment.^(10)^

Although OFDAs offer a variety of options, the meals ordered through these apps, like many takeout meals, tend to be large servings of foods that are often high in saturated fat, sugar, and sodium.^(11)^ Moreover, the unhealthy environment made readily available on these applications, with most of the choices being fast-food restaurants, encourages consumers to order and consume food more frequently. Eating meals away from home, including those ordered through food delivery apps, has been linked to higher body mass index (BMI) and increased risk for cardiometabolic health issues.^(12)^ Although there is limited research on the effects of food delivery apps on nutrition or health, there are concerns that regular use of these apps may lead to increased consumption of nutrient-poor, energy-dense foods.^(13)^ Studies evaluating the healthiness of popular food outlets and the nutritional quality menu items on OFDAs revealed that menus consisted of predominantly unhealthy choices and healthier alternatives were priced higher.^(14–16)^ Moreover, the effects of frequent use of OFDAs are likely to extend beyond excessive weight gain to include negative impacts on physical activity levels and excessive waste similar to offline consumption of meals away from home.^(2)^

While many individuals are becoming more conscious about their diet and trying to make dietary changes, research suggests that consumers have a limited understanding of what constitutes a healthy diet.^(17)^ According to the World Health Organization (WHO), a healthy diet includes a variety of fruits, vegetables, whole grains, lean protein sources, and healthy fats, while limiting the intake of saturated and trans fats, added sugars, and salt.^(18)^ However, consumers willing to make healthy food choices may encounter a scarcity of such options within OFDAs.^(19)^ Moreover, the healthiness of food goes beyond the variety of nutrients it provides and also includes aspects of food safety such as proper preparation and handling.^(20)^ Information about employee hygiene, food handling, and the cleanliness of the establishment is not typically provided to consumers when ordering through OFDAs.^(21)^ As a result, it is unlikely that consumers who order food online are aware of the hygienic status of the restaurant, or the food being prepared. However, perception can dictate what individuals consider to be nutritious, safe, and accessible. Perception can be influenced by various factors, including societal norms, cultural beliefs, and most importantly marketing strategies employed by food industry stakeholders.^(22)^ For instance, marketing certain foods as ‘healthy’ or ‘natural’ within online food apps can impact consumers’ perceptions of their nutritional value. Consequently, it is essential to understand how healthy food availability is perceived on online platforms, to promote informed dietary choices and address potential misconceptions.

Young adults have the highest proportion of usage of food retail apps and are capable of making independent eating and spending decisions, making them a population of interest.^(13,23)^ Given the autonomy and poor eating habits demonstrated by university students,^(24)^ it is expected that this population will exhibit a high frequency of utilizing OFDAs and make less healthy meal choices. Moreover, when considering the increasing prevalence of overweight and obesity among young adults (33.5%) in the UAE,^(25)^ the effects of these OFDAs could be of great concern. Considering the above-mentioned concerns, this research aimed to address the gaps in knowledge by assessing the use of OFDAs among university students in the UAE and the perception of healthy and safe food availability in OFDAs.

## Methods

### Study design and sampling

A cross-sectional, web-based study was conducted between January and March 2022 targeting university students in the UAE. The inclusion criteria were students enrolled at any university in the UAE, aged 18 years and above, and who use OFDAs (≥1 time/month). Convenient and snowball sampling methods were used to recruit participants and ensure large-scale distribution and recruitment. The minimum sample size was calculated based on the following equation with a confidence interval of 99%:

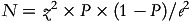




where z = 2.576; P = (estimated proportion of the population that presents the characteristic) = 0.5; e (margin of error) = 0.05; N (sample size) = 664 participants, plus 20% (attrition rate) = 797 participants.

Students were invited to participate through a web link connecting to the online survey on Google Forms which was shared via social media platforms (Instagram™, WhatsApp™, and Facebook™). Participants were also encouraged to share the questionnaire’s URL with their friends and family members. The online survey included an information sheet along with a consent form on the first page. Participants had to read the study objectives and consent to take part in the research before accessing the questions. Participants were also informed that their participation was voluntary, that they could exit the survey at any point, and that their responses were completely anonymous. Screening questions were also available on the first page of the survey to ensure that participants met the inclusion criteria. Consenting participants were then directed to the survey questions which required 5–10 min to fill out and submit. The study obtained ethical approval from the University of Sharjah Research Ethics Committee (REC-22-02-16-09-S). All participants provided written informed consent.

### Questionnaire design

The present study utilized a data collection tool developed by the researchers upon comprehensive examination of relevant literature on OFDAs use, availability of healthy food options on apps, and consumer perceptions.^(26–28)^ The initial version of the survey instrument consisted of 35 closed-ended questions utilizing Likert-scale, dichotomous, multiple choice, and checklist format. The tool was evaluated by a panel of four experts in the field of nutrition and food safety with a substantial background in survey research in areas of food safety, nutrition, and public health. The panel members were asked to evaluate the questions based on language, clarity, repetition, content, and relevance to the objectives of the study, with any question receiving an average score of less than 70% being amended or removed. Additionally, any other suggestions from the reviewers were taken into consideration. The survey’s reliability was evaluated using Cronbach’s alpha coefficient for internal consistency, yielding a value of (0.751), indicating an acceptable level of internal consistency. The survey instrument was then translated from English to Arabic and back-translated to English by three bilingual experts to ensure the accuracy of the translation. The final version of the survey was created using Google Forms, a survey administration software included in the Google Drive office suite. The survey was pilot-tested with 20 individuals and no further revisions were required. Data from the pilot test were not included in the final analysis of the study.

The final version of the survey consisted of four sections including a total of 23 close-ended questions. The socio-demographic section inquired about sex, age, university location (Emirate), living situation, and monthly allowance. The second section included questions on OFDAs usage. This inquired about the most frequently used food apps, the type of food ordered through these apps, factors affecting food choices, and whether participants considered healthy options when ordering food. The next sections investigated the consumers’ perception of a healthy meal and concerns regarding ordering healthier food alternatives. It also assessed the participants’ perceptions of healthy food availability on OFDAs. The final section focused on food safety in OFDAs. The questions inquired about the consumers’ perception of the cleanliness of the food delivered through OFDAs, the influence of meal packaging on food choices, and the impact of meal temperature during delivery on food safety and quality. The answer option for the food healthiness and safety perception statements was a five-point Likert scale ranging from strongly disagree to strongly agree.

### Data analyses

Descriptive analysis for categorical data was expressed as counts and percentages. The frequency of using OFDAs was categorized into a dichotomous variable where; frequent use corresponds to response options: daily, 4–6 times/week, and 2–3 times/week; infrequent use corresponds to response options: once/week and once/month. Cross tabulations and chi-square tests were used to determine the association between categorical variables with OFDAs usage. Assumptions of normality and equal variances were violated thus non-parametric statistical methods were used. To investigate potential differences in perceptions among distinct demographic groups, the Mann–Whitney U test was employed for pairwise comparisons, while the Kruskal–Wallis H test was utilized to assess variations across multiple independent groups and perception statements. Data were analyzed using SPSS software, version 26.0 (SPSS, Chicago, IL, USA). A P-value of <0.05 was considered statistically significant.

## Results

### Socio-demographic characteristics and OFDAs use

A total of 1096 university students participated in the study. Table 1 presents the socio-demographic characteristics of the participants. Almost half of the participants were 21–23 years old (46.8%). More females than males completed the online survey (64.5%). Most of the participants were enrolled at universities in Sharjah (39.6%) followed by Dubai (28.1%) and Abu Dhabi (21.0%). Most participants reported living with their families (81.5%) and had a monthly allowance of >5000 AED (39.1%). Most of the participants reported using OFDAs 2–3 times per week and at least once per week (30.0% and 27.0% respectively) (data not shown). Almost half of the participants were frequent users of food applications (51.95%). The Chi-square analysis revealed that older participants (P = 0.004), those who live in the university dorms (P < 0.001), and those with higher monthly allowances (P < 0.001) were using the OFDAs more frequently.

**Table 1.** Sociodemographic characteristics and usage trends of OFDAs (*n* = 1096)

		Frequency of using OFDAs		P-value^*^
	Total n (%)	Frequent n (%)	Infrequent n (%)	
Total		569 (51.9)	527 (48.1)	
Age (years)				
18–20	412 (37.6)	190 (46.1)	222 (53.9)	0.004
21–23	513 (46.8)	276 (53.8)	237 (46.2)	
24–27	171 (15.6)	103 (60.2)	68 (39.8)	
Sex				
Females	707 (64.5)	356 (50.4)	351 (49.6)	0.09
Males	389 (35.5)	213 (54.8)	176 (45.2)	
University location				
Abu Dhabi	230 (21.0)	125 (54.3)	105 (45.7)	0.42
Dubai	308 (28.1)	152 (49.4)	156 (50.6)	
Sharjah	434 (39.6)	233 (53.7)	201 (46.3)	
Northern Emirates^a^	124 (11.3)	59 (47.6)	65 (52.4)	
Living situation				
With family	893 (81.5)	429 (48.0)	464 (52.0)	<0.001
University dorms	203 (18.5)	140 (69.0)	63 (31.0)	
Monthly allowance				
<1000 AED	402 (36.7)	155 (38.6)	247 (61.4)	<0.001
1000 – <5000 AED	266 (24.3)	178 (66.9)	88 (33.1)	
>5000 AED	428 (39.1)	236 (55.1)	192 (44.9)	
Factors influencing food choice^b^				
Food appearance	720 (65.7)	387 (53.8)	333 (46.3)	0.05
Price	855 (78.0)	442 (51.7)	413 (48.3)	0.42
Time of delivery	554 (50.5)	305 (55.1)	249 (44.9)	0.02
Healthy options availability	221 (20.2)	127 (57.5)	94 (42.5)	0.04
Display nutrient/calorie content	141 (12.9)	76 (53.9)	65 (46.1)	0.34
Hygienic status	532 (48.5)	291 (54.7)	241 (45.3)	0.04

a Northern Emirates including Ajman, Um Al Quwain, Ras Al Khaimah, and Fujairah.

b Multiple responses were allowed, and data presented for those who responded ‘yes’.

* P-value was based on the chi-square test at a 5% level.

**Fig. 1.**
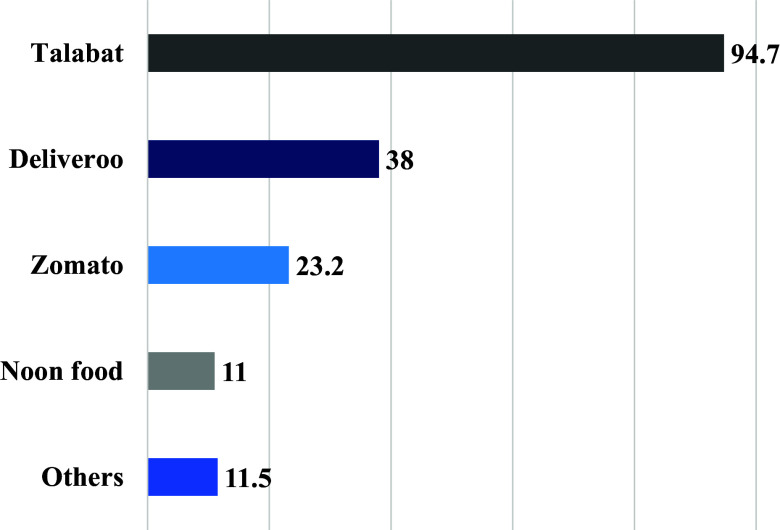
Most OFDAs used (n = 1096). (Multiple responses were allowed; others include Uber Eats, Eat Clean me, EatEasy, Careem now, Instashop, Carriage, and restaurant apps).

**Fig. 2.**
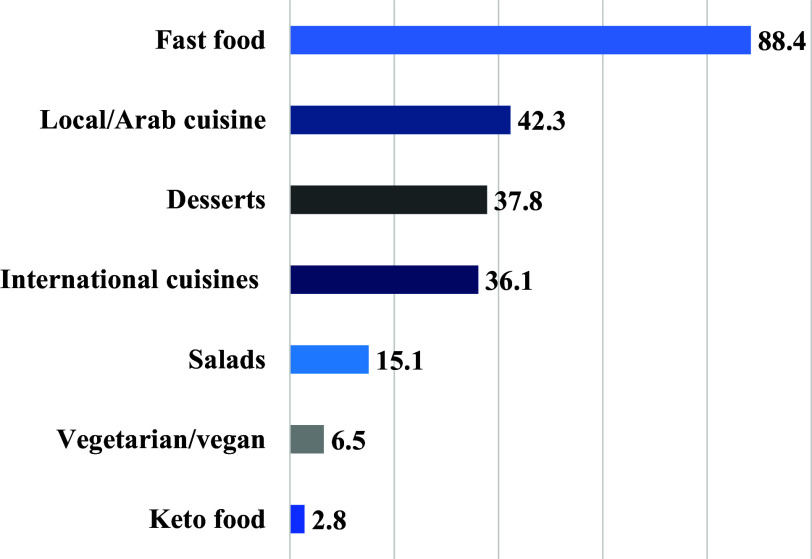
Most ordered cuisine (n = 1096). (Multiple responses were allowed).

The factors that mostly influenced the participants’ food choices, were affordability (78.0%) and appearance (65.7%) (Table 1). About two-thirds of the participants reported looking for healthy food options when ordering through OFDAs (69.3%). The most used OFDAs was Talabat (94.6%) (Fig. 1), and the most popular food choice was fast food (88.4%) (Fig. 2).

### Perceptions of healthy food on OFDAs

**Fig. 3.**
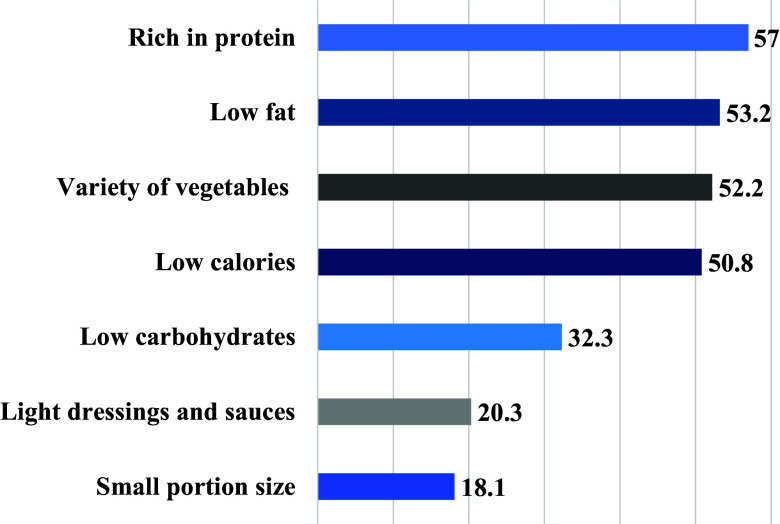
Perception of a healthy meal when using OFD applications among participants (n = 1096). Multiple responses were allowed.

**Fig. 4.**
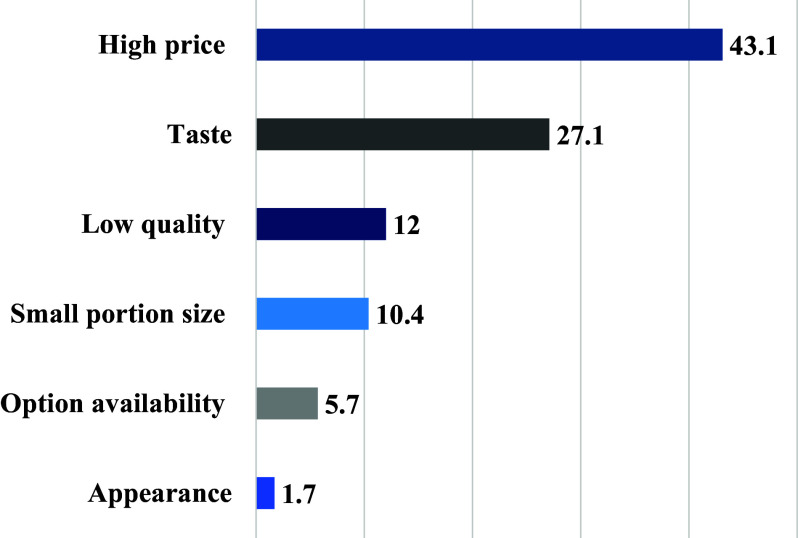
Concerns about ordering a healthy food choice among participants (n = 1096).

When participants were asked about what they think constitutes a healthy meal around half of them reported a meal that is rich in protein, low in fat, has a variety of vegetables, and is low in calories (50.8%–57.0%) (Fig. 3). Barriers to ordering healthy foods included affordability (43.1%), taste (27.1%), and quality (12%) (Fig. 4).

**Fig. 5.**
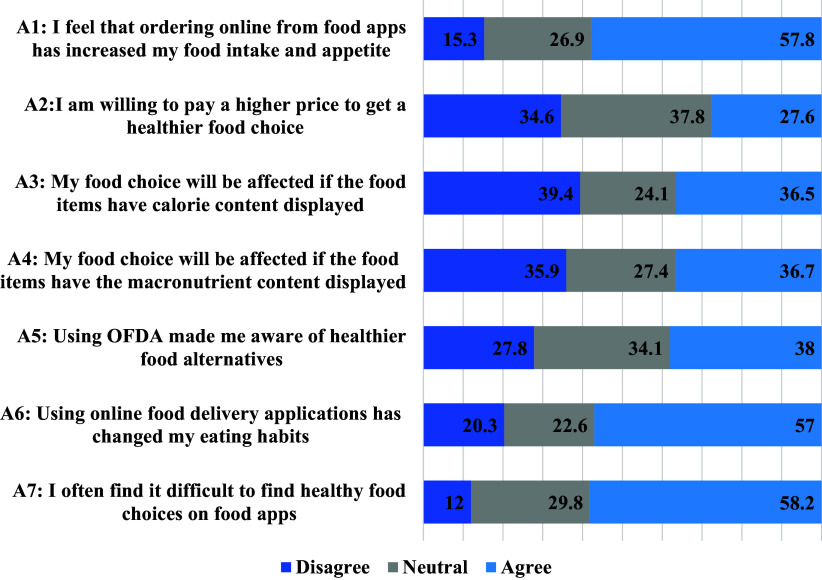
Perceptions about healthy food ordering through the OFD applications (n = 1096).

Figure 5 presents participants’ agreement on seven statements related to placing healthy food orders through OFDAs. Further analysis of the association of different socio-demographic variables and perception statements is presented in Table 2. Around 60% of participants agreed that the OFDAs increased their food intake and appetite. Further analysis revealed that those with higher allowances and who used OFDAs more frequently agreed more with this statement than their counterparts (P < 0.001). Additionally, 60% of participants agreed that their eating habits were affected by OFDAs, specifically in terms of consuming more late-night snacks or eating alone. This was found to be significantly different among participants based on whether they lived with the family, their university location, and their allowance (P < 0.001, P = 0.006, P < 0.001 respectively). A similar proportion of participants (58.2%) agreed on the difficulty of finding healthy food options, however, this did not differ among different groups. Additionally, 37% of participants agreed that having calorie and macronutrient content displayed on OFDAs might affect their food choices, and those with higher allowances were more in agreement with this statement than their counterparts (P = 0.02). Furthermore, 38% of participants agreed that OFDAs made them aware of healthier food alternatives, and this was found to be significantly different based on whether they lived with family or not, and their university location (P = 0.04, P = 0.047). Only 27.6% of participants expressed willingness to pay higher prices for healthier food options on OFDAs, which was found to be significantly different based on their allowance (P = 0.001).

**Table 2.** Socio-demographic effects on participants’ perceptions about healthy food ordering through OFDAs

	Mann-Whitney U^*^			Kruskal-Wallis H^*^		
Item^a^	Sex	Living with family	Frequency of use	Age	University location	Allowance
A1	0.36	0.15	<0.001	0.39	0.46	<0.001
A2	0.65	0.15	0.27	0.98	0.66	0.001
A3	0.05	0.05	0.08	0.31	0.73	0.02
A4	0.23	0.11	0.23	0.23	0.98	0.19
A5	0.05	0.04	0.05	0.23	0.05	0.24
A6	0.18	<0.001	<0.001	0.88	0.006	<0.001
A7	0.26	0.46	0.13	0.35	0.44	0.17

a A1: I feel that ordering online from food apps has increased my food intake and appetite; A2: I am willing to pay a higher price to get a healthier food choice; A3: My food choice will be affected if the food items have calorie content displayed; A4: My food choice will be affected if the food items have the macronutrient content displayed; A5: Using OFDAs made me aware of healthier food alternatives; A6: Using online food delivery applications has changed my eating habits; A7: I often find it difficult to find healthy food choices on food apps.

* Significance level at P < 0.05.

### Perceptions of food safety and delivery hygiene

**Fig. 6.**
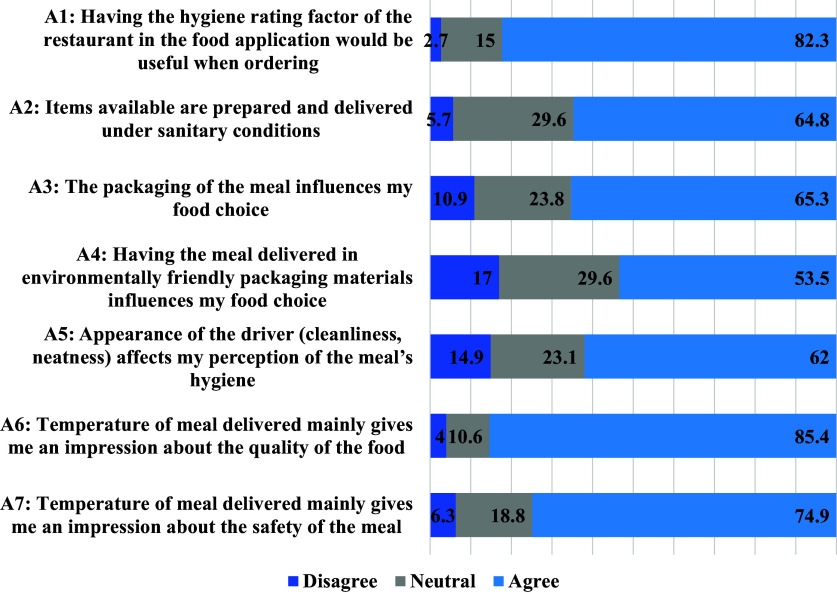
Perceptions about food safety and hygiene while ordering through the online food applications (n = 1096).

Participants were asked to rate their agreement to several statements related to food safety and hygiene of food delivery as presented in Fig. 6. Further analysis of the association of different socio-demographic variables and perception statements is presented in Table 3. Of the participants, 82.3% believed that hygiene ratings would be useful when ordering food, which did not differ between groups. Additionally, 64.8% of participants believed that food available through the OFDAs was prepared and delivered under sanitary conditions, with those with higher allowance having a stronger agreement (P = 0.03). Furthermore, 65.3% of participants agreed that packaging influences their food choice with certain subgroups such as females, frequent users, older participants, and those with higher allowance showing higher agreement (P = 0.04, P = 0.005, P = 0.0005, P = 0.05, and P = 0.009 respectively). Approximately half of the participants (53.5%) agreed that the use of environmentally friendly packaging influences their food choice and 62.0% agreed that the driver’s cleanliness and neatness had an impact on their perception of the meal’s hygiene. Additionally, 85.4% and 74.9% of participants respectively believed that the temperature of the meal upon delivery is a good indicator of both the quality and safety of the food with frequent users having a stronger agreement on the indicator of quality (P = 0.03), and those with higher allowance having a stronger agreement on the indicator of safety (P < 0.001).

**Table 3.** Socio-demographic effects on participants’ attitudes toward food safety and delivery hygiene while ordering through OFDAs

	Mann-Whitney *U* ^*^			Kruskal-Wallis *H* ^*^		
Item^a^	Sex	Living with family	Frequency of use	Age	University location	Allowance
A1	0.37	0.23	0.85	0.16	0.22	0.44
A2	0.18	0.31	0.17	0.49	0.50	0.03
A3	0.04	0.67	0.005	0.05	0.47	0.009
A4	0.15	0.13	0.17	0.40	0.15	0.35
A5	0.85	0.29	0.07	0.53	0.04	0.14
A6	0.15	0.80	0.03	0.57	0.92	0.09
A7	0.54	0.63	0.06	0.06	0.06	<0.001

a A1: I believe that having the hygiene rating factor of the restaurant in the food application would be useful when ordering; A2: I believe that the items available are prepared and delivered under sanitary conditions; A3: I believe that the packaging of the meal influences my food choice; A4: I believe that having the meal delivered in environmentally friendly packaging materials influences my food choice; A5: The appearance of the driver (cleanliness, neatness) affects my perception of the meal’s hygiene; A6: The temperature of the meal when delivered mainly gives me an impression about the quality of the food; A7: The temperature of the meal when delivered mainly gives me an impression about the safety of the meal.

* Significance level at P < 0.05.

## Discussion

The current study explored the use of OFDAs and the perception of food healthiness and safety among university students in the UAE. The findings showed that over half of university students used OFDAs at least 2–3 times per week, with Talabat emerging as the most popular application used among participants. The use of OFDAs has been rapidly increasing globally, and this trend is also observed in the UAE.^(29)^ The frequency of OFDAs use in the current study is slightly higher than that of the Brazilian population and college students in Malaysia, as most participants in these studies used OFDAs 1–3 times per month.^(27,30)^ Talabat is an online food ordering service that allows customers to find local restaurants, browse menus, and place orders with options for online payment or cash on delivery. Founded in 2004 in Kuwait, Talabat has grown to become a key player in the Arab region and is one of the leading OFDAs in the UAE.^(31)^ Other applications were less popular in the current study compared to Talabt, which could be due less variants of restaurants or limited delivery coverage areas.

The present findings revealed that older age, living away from family, and higher allowance were associated with higher use. This agrees with recent research indicating that the demographic profile of a typical online food consumer is more likely to be young, well-educated, living in a small family unit, with a very good or adequate overall economic condition.^(32)^ Moreover, it has been suggested that consumers may be less likely to continue using OFDAs if they do not receive approval from their friends and family.^(33)^ Therefore, it would be expected that the absence of social influence from family members when living in university dorms will increase the potential use of these apps.

The framework for understanding the physical food environment includes both external and internal factors that can also be applied to the digital realm in which OFDAs operate.^(7)^ As for internal factors, price and food appearance were the most significant factors influencing participants’ food choices when using OFDAs, while factors such as healthy food availability and display of nutrition information were of lesser influence. This is a rather expected find as university students are more likely to prefer options that are both visually appealing and budget-friendly. The findings of this study also align with previous research which found that university students tend to prioritize convenience, time availability, sensory appeal, and food price over the nutritional value of food.^(34)^ Additionally, another study suggested that even though university students may know nutritional requirements, they may not necessarily make healthy food choices.^(35)^ This suggests that interventions aimed at promoting healthy food choices among university students should focus on making healthy options more accessible and affordable.

The present study also found differences in the importance of certain factors between frequent and infrequent OFDAs users. Specifically, time of delivery, availability of healthy options, and hygienic status of the restaurant were more important to frequent consumers. Ordering meals through OFDAs can save a significant amount of time compared to grocery shopping, cooking, and cleaning. This explains why many people have become dependent on and frequently use OFDAs.^(36)^

As for external influences, OFDAs have made it possible for consumers to access a wider variety of food options by expanding the availability of food service outlets in the physical environment. Moreover, social media marketing powers the food industry with many businesses using various employ a variety of marketing tactics, including the use of visual media, hyperlinks, incentives, and promotional giveaways to attract consumers.^(37)^ However, it is important to note that the majority of food marketing activities do not promote healthy food choices and instead, tend to advocate for unhealthy options.^(38)^ This reflects highly on the present findings as using OFDAs to order fast food was the most prominent response among the participants. A study of food outlets available in China indicated that there were four times more unhealthy food options available compared to healthy options.^(39)^ Additionally, out of the total food outlets that offered food delivery services, fast-food restaurants made up a significant portion of 65.5%.^(39)^ This makes it more likely for individuals to be exposed to unhealthy food choices in fast-food settings. Moreover, the trend in the present study can be also explained in the context of the nutrition transition, and the shift from a traditional diet to more Westernized, energy-dense food consumption.^(40)^ The existing literature indicates that the predominant food outlets featured on OFDAs tend to offer food options that are classified as unhealthy. Additionally, existing literature indicates that the predominant food outlets featured on OFDAs tend to offer food options that are classified as unhealthy. Moreover, the most sought-after menu items on these platforms often consist of discretionary foods, emphasizing a trend that may contribute to the accessibility and consumption of nutritionally poor-quality foods.^(14,16)^ Overall, the availability of unhealthy food restaurants can play a role in the development of overweight and obesity among young adults. The rise of OFDAs caters to consumer preferences for simple and convenient options. Yet, this surge seems to encourage the purchase of nutritionally poor foods, calling for urgent and substantial attention within public health nutrition strategies and policies. Efforts should be made to increase access to healthy food options in areas with a high density of fast-food restaurants and to promote healthier food choices through targeted marketing and educational campaigns.

Understanding how OFDAs users perceive healthy foods is essential in designing effective interventions aiming at improving their diet quality.^(41)^ In the present study, around two-thirds of the participants reported considering healthy food options while ordering. Findings from a study among Malaysian students indicated that although participants had negative perceptions of the availability of healthy options, they were eager to purchase healthy food in the future.^(27)^ Moreover, half of the participants in the present study reported a meal that is high in protein, low in fat, includes a variety of vegetables, and low in calories as healthy. These findings are in line with the most recent dietary guidelines emphasizing that a healthy meal should be balanced, of high nutrient density, and within the individual’s daily calorie needs.^(42)^ Further, the present findings revealed that participants were mainly concerned about the price of healthy food on OFDAs. This is rather expected due to the fairly lower cost of unhealthy food options compared to healthier ones^(43)^ and evidence indicating that healthy food can cost up to twice the amount of unhealthy food.^(44)^ The role of digital marketing on OFDAs and providing consumers with discounts exacerbates their preference to choose those items despite their dietary quality. This highlights the importance of diminishing the obstacles to healthy eating among the public.^(45)^

The current study also highlighted participants’ perceptions of healthy food ordering through OFDAs. Most participants agreed that OFDAs increased their appetite, food intake, and eating habits such as more snacking and eating alone. Additionally, most participants agreed on the difficulty of finding healthy options. A lesser proportion of the participants thought that having nutrition information available would affect their purchasing decisions and expressed willingness to pay higher prices for healthier food options. These perceptions appeared to be influenced by factors such as allowance, frequency of use, and living with family or not. Unfortunately, the impact of the increased use of OFDAs on public health remains unclear. While OFDAs do not have a direct impact on their frequent users’ health and nutrition status, they still play an indirect role by providing easy access to mostly unhealthy food options.^(21)^ In a recent Canadian study, 759 menu items were assessed using food and nutrient databases and revealed low healthy eating index scores indicating that menu items do not align with healthy eating recommendations.^(46)^ With that, frequent OFDAs users are more exposed to imbalanced diets and more prone to consume high amounts of saturated fat, added sugars, and sodium. Moreover, it is important to pay attention to both the size of the servings and the nutritional information provided. A study in the UK has shown that meals consumed outside of the home or as takeout typically contain double the amount of calories compared to products sold at retail stores.^(47)^ These patterns consequently may contribute to higher risks of non-communicable diseases.^(48)^ Restaurant menu calorie labeling has emerged as a cost-effective strategy that can influence the food environment and increase consumer awareness.^(49,50)^ Although not typically implemented on OFDAs, some healthy food outlets on those apps display nutrition information in the UAE. A wider implementation is observed in Saudi Arabia which mandated all digital and non-digital food services to display nutrition information on menus.^(51)^

Ensuring the safety of food is a collective effort among all parties involved in the food industry. As the utilization of OFDAs becomes more prevalent, it is imperative to pay particular attention to potential risks related to the preservation, transport, and delivery of food.^(21)^ In the present study, participants mostly believed that hygiene ratings would be useful when ordering food. This finding is in line with previous research which suggests consumers were more likely to place an order from a restaurant with high hygiene ratings.^(52)^ Moreover, another study revealed that customers were less likely to revisit a restaurant they considered to be unhygienic.^(53)^ Although the hygiene rating on OFDAs is not applied in the UAE, the government and municipalities are adamant about ensuring food safety across all food service establishments.^(54)^ This provides consumers with a sense of assurance when ordering through OFDAs. Moreover, by referring to other consumers’ comments, reviews, and feedback, consumers can be informed of any unfavorable food hygiene or mishandling.^(52)^ From a public health perspective, the implementation of mandatory hygiene rating posting, when executed correctly, is a viable public policy that promotes transparency, improves public health, and empowers consumers to make informed choices.^(55)^ Our findings indicated that most participants believed that the packaging of their meals and environmentally packaged meals influence their food choices. This agrees with relevant literature indicating that consumers tend to prefer meals packaged with higher quality materials, as they perceive them to be reflective of the quality of the meal contained within it.^(56)^ Additionally, customer perceptions are influenced by various factors, including the cleanliness of the food packaging and the condition of the received meal.^(57)^ Favorably, the findings of the study are suggestive that environmentally-friendly packaging is becoming an important consideration for consumers when choosing food options.^(58)^ In this study, it was found that meals delivered hot were perceived as meals of higher quality and safety. Research indicates that consumers are concerned about food safety when eating out and pay attention to various attributes such as observed cleanliness, the appearance of staff, temperature of food, and general impression of the restaurant.^(59,60)^

The WHO is currently investigating digital food environments as part of its program for promoting healthy and sustainable diets and the prevention of NCDs.^(61)^ Despite the potential unfavorable effects of OFDAs on consumers’ health and nutrition status, it is suggested that these digital platforms can be employed to promote healthy eating habits and food options and reduce the risk of NCDs.^(21)^ The present study is the first to investigate university students’ use and perceptions of healthy food on OFDAs in the region. The findings present insights into perceptions of healthy food ordering among users of these apps, providing opportunities for future research and interventions to enhance consumers’ awareness and understanding of healthy and safe food choices. However, this study is subject to some limitations. The cross-sectional study design may affect the generalizability of the results and the ability to infer causality. Moreover, the sampling method may be susceptible to nonresponse bias. Lastly, the use of a self-reported questionnaire is subject to bias such as social desirability bias or recall bias, which can affect the accuracy of the participant’s responses.

## Conclusion

The current study found that convenience and affordability are important factors that influence food choices. The study highlights the need for OFDAs to provide more affordable and appealing healthy food options to meet the demands of consumers and suggests that improved nutrition information and hygiene standards could help to promote healthy food choices among university students. The findings can inform future interventions and policies aimed at promoting healthier food choices among university students in the UAE, by making healthy options more accessible and affordable through OFDAs. Future research can include investigating strategies to increase the availability and appeal of healthy food options on OFDAs, examining the potential impact of these strategies on overall public health, and investigating the role of OFDAs on food choices and eating habits. Currently, some OFDAs have started to include information about the nutritional content of the foods that are being offered and have also started to offer healthy food options such as salads and vegetable dishes. However, more research is needed to understand the impact of these initiatives on consumer behavior and the extent to which they are effective in promoting healthy food choices.

## Abbreviations

**BMI:** body mass index; **OFDAs:** Online food delivery applications; **UAE:** United Arab Emirates; **WHO:** World Health Organization.

## Supplementary material

The supplementary material for this article can be found at https://doi.org/10.1017/jns.2024.21

## Supporting information

Cheikh Ismail et al. supplementary material Cheikh Ismail et al. supplementary material

## Data Availability

The datasets used and/or analyzed during the current study are available from the corresponding author on reasonable request.
