# Supplementary material for: A cross-sectional study on online food delivery applications (OFDAs) in the United Arab Emirates: use and perceptions of healthy food availability among university students
Source: J Nutr Sci. 2024 Oct 10;13:e62. doi: 10.1017/jns.2024.21 (PMC11503857; doi:10.1017/jns.2024.21)
Supplement: Cheikh Ismail et al. supplementary material [file S2048679024000211sup001.docx]

**Online Food Delivery Applications (OFDAs) in the United Arab Emirates: Use and Perceptions of Healthy Food Availability among University Students**

# **Socio-demographic characteristics**

1. **Please specify your gender**

- Male
- Female

1. **Please specify your age ------- (years)**
2. **University Location?**

- Abu Dhabi
- Dubai
- Sharjah
- Other emirates

1. **Where do you live?**

- With family
- University dorms
- Alone

1. **Approximately, what is your monthly income/ allowance**

- Under 1000 AED
- 1000-<5000 AED
- >5000 AED

# **Use of online food delivery applications**

1. **Do you use online food delivery applications?**

- Daily
- 4-6 times/ week
- 2-3 times/ week
- Once a week
- Once a month
- Never (leave the survey)

1. **Which online food delivery application do you usually use the most? (Select all that applies)**

- Zomato
- Talabat
- Deliveroo
- Uber Eats
- Noon food/ Now Now
- Eat Clean Me
- EatEasy
- Careem now
- Instashop
- Other, specify …….

1. **What cuisine do you mostly look for when using food applications?**

- Vegetarian / vegan
- Fast food
- International cuisine
- Local/Arab cuisine
- Salads
- Keto food
- Desserts

1. **Which of the following mostly influences your food choice when using online food delivery applications?**

- Food appearance
- Price
- Time of delivery
- Healthy options availability
- Display nutrient and calorie content
- Hygienic status of the restaurant

1. **Do you look for healthy for healthy food options when ordering through OFDAs?**

- Yes
- No

# **Healthy choices on food delivery applications**

|  | **Strongly**  **agree** | **Agree** | **Neutral** | **Disagree** | **Strongly**  **disagree** |
| --- | --- | --- | --- | --- | --- |
| I often find it difficult to find healthy food choices on food apps |  |  |  |  |  |
| Using online food delivery applications has changed my eating habits |  |  |  |  |  |
| Using OFDA made me aware of healthier food alternatives |  |  |  |  |  |
| My food choice will be affected if the food items have the macronutrient content displayed |  |  |  |  |  |
| My food choice will be affected if the food items have calorie content displayed |  |  |  |  |  |
| I am willing to pay a higher price to get a healthier food choice |  |  |  |  |  |
| I feel that ordering online from food apps has increased my food intake and appetite |  |  |  |  |  |

# **Food Safety & food delivery applications**

|  | **Strongly**  **agree** | **Agree** | **Neutral** | **Disagree** | **Strongly**  **disagree** |
| --- | --- | --- | --- | --- | --- |
| Temperature of meal delivered mainly gives me an impression about the safety of the meal |  |  |  |  |  |
| Temperature of meal delivered mainly gives me an impression about the quality of the food |  |  |  |  |  |
| Appearance of the driver (cleanliness, neatness) affects my perception of the meal’s hygiene |  |  |  |  |  |
| Having the meal delivered in environmentally friendly packaging materials influences my food choice |  |  |  |  |  |
| The packaging of the meal influences my food choice |  |  |  |  |  |
| Items available are prepared and delivered under sanitary conditions |  |  |  |  |  |
| Having the hygiene rating factor of the restaurant in the food application would be useful when ordering |  |  |  |  |  |
